# The role of imaging in outcomes and safety monitoring for preclinical intervention studies

**DOI:** 10.1002/alz.085974

**Published:** 2025-01-09

**Authors:** Michael Rafii, Prashanthi Vemuri

**Affiliations:** ^1^ University of Southern California, San Diego, CA USA; ^2^ Alzheimer’s Therapeutic Research Institute, San Diego, CA USA; ^3^ Mayo Clinic, Rochester, MN USA

## Abstract

Recent breakthrough findings in clinical trials on amyloid‐lowering therapies have led to the approval of these drugs for the treatment of amyloid‐ positive elderly individuals who show symptoms of mild cognitive impairment and mild dementia. The next frontier is the testing the efficacy of treatments for secondary prevention of AD dementia. Phase III trials in asymptomatic AD are already under way, raising a host of novel questions on the sequelae of trial participation such as the emotional and social repercussions of biomarker disclosure, understanding the risk of side effects and eventually weighing the risk‐benefit ratio of amyloid‐lowering treatment. In this session jointly organized by the Neuroimaging and Partnering with Research Participants PIAs, I will focus on the participants’ perspective on the role of imaging in outcomes and safety monitoring of preclinical interventional studies. I will outline the importance of a partnership between the study team and participants in the design and conduct of study procedures to ensure that they are both efficient and ethical.

